# Why Molnupiravir Fails in Hospitalized Patients

**DOI:** 10.1128/mbio.02916-22

**Published:** 2022-11-14

**Authors:** Ashley N. Brown, Yinzhi Lang, Jieqiang Zhou, Evelyn J. Franco, Kaley C. Hanrahan, Juergen B. Bulitta, George L. Drusano

**Affiliations:** a Institute for Therapeutic Innovation, University of Florida, Orlando, Florida, USA; b Department of Medicine, College of Medicine, University of Florida, Orlando, Florida, USA; c Department of Pharmaceutics, College of Pharmacy, University of Florida, Orlando, Florida, USA; d Department of Pharmacotherapy and Translational Research, College of Pharmacy, University of Florida, Orlando, Florida, USA; MedImmune

**Keywords:** antivirals, hollow fiber infection model, molnupiravir, SARS-CoV-2, pharmacodynamics

## Abstract

Severe acute respiratory syndrome coronavirus 2 (SARS-CoV-2), the causative agent of coronavirus disease 2019 (COVID-19), has radically altered daily life. Effective antiviral therapies to combat COVID-19, especially severe disease, remain scarce. Molnupiravir is an antiviral that has shown clinical efficacy against mild-to-moderate COVID-19 but failed to provide benefit to hospitalized patients with severe disease. Here, we explained the mechanism behind the failure of molnupiravir in hospitalized patients and identified alternative dosing strategies that would improve therapeutic outcomes in all patients with COVID-19. We showed that delaying therapy initiation markedly decreased the antiviral effect of molnupiravir, and these results were directly related to intracellular drug triphosphate pools and intracellular viral burden at the start of therapy. The adverse influence of therapeutic delay could be overcome by increasing drug exposure, which increased intracellular molnupiravir triphosphate concentrations that inhibited viral replication. These findings illustrated that molnupiravir must be administered as early as possible following COVID-19 symptom onset to maximize therapeutic efficacy. Higher doses may be effective in patients hospitalized with severe disease, but the safety of high-dose molnupiravir regimens is unknown. Our findings could be extended to design effective regimens with nucleoside analogs for other RNA viruses, especially those with pandemic potential.

## INTRODUCTION

Severe acute respiratory coronavirus 2 (SARS-CoV-2), the RNA virus that is the causative agent of coronavirus disease 2019 (COVID-19), has radically changed modern society by decimating economies, overwhelming healthcare systems, and forcing entire populations of people into lockdown and isolation. These conditions were exacerbated by significant morbidity and mortality rates associated with infection. The rapid production of safe and effective vaccines for SARS-CoV-2 has helped to change the course of the pandemic, providing high levels of protection against severe disease and death in the fully vaccinated (1, 2). However, vaccine hesitancy (3–5) and immune escape by SARS-CoV-2 variants (6–8) demonstrate some of the challenges associated with vaccination and highlight the gaps left by the vaccine as well as monoclonal antibody treatments. Antiviral agents are essential to fill these therapeutic gaps to control the ongoing COVID-19 pandemic.

Historically, antivirals have played a pivotal role in the management of several viral diseases, including human immunodeficiency virus (HIV), hepatitis C virus (HCV), and influenza virus (9–12). Nucleoside/nucleotide polymerase inhibitors are a class of drugs that have demonstrated activity against a wide variety of viruses and often serve as the backbone for both HIV and HCV combination therapeutic regimens (13–15). These agents are frequently considered the first line of defense for emerging viral infections (i.e., SARS-CoV-2) due to their broad-spectrum activity and high genetic barrier to resistance (15–18). Consequently, nucleoside/nucleotide analogs, including favipiravir, remdesivir, and galidesivir have all been examined to combat COVID-19 and yielded various degrees of efficacy (19–21). Therapeutic outcomes with these agents have been uniformly disappointing in hospitalized patients, but some have shown promising results in preventing severe disease and hospitalization in outpatients who received treatment early in infection (20, 22, 23). These trial results illustrate the importance of early treatment initiation for nucleoside/nucleotide antivirals to maximize efficacy against COVID-19. To initiate antiviral therapy early, orally available agents are essential because they allow patients to self-medicate shortly after symptom onset and documentation of infection without the need for a clinical setting.

Molnupiravir is a new nucleoside polymerase inhibitor with good oral bioavailability that has demonstrated potent antiviral activity against SARS-CoV-2 (24). Clinical trial results revealed that 800 mg molnupiravir twice daily reduces the risk of hospitalization or death in outpatients who received the early therapeutic intervention (MOVe-OUT Trial ClincialTrials registration number NCT04575597) (25). However, molnupiravir failed to improve clinical outcomes in hospitalized patients (MOVe-IN Trial ClinicalTrials registration number NCT04575584) (26). Here, we sought to investigate the mechanism behind the disparate conclusions between the inpatient and outpatient trials. In turn, we also sought to identify alternative dosing strategies for molnupiravir that hold promise to improve therapeutic outcomes in patients infected with SARS-CoV-2.

## RESULTS

### Antiviral potency of nucleoside/nucleotide analogs against SARS-CoV-2.

Upon oral administration, the molnupiravir prodrug is rapidly converted to EIDD-1931 (β-_D_-N4-hydroxycytidine [NHC]) in human plasma (27). EIDD-1931 is systemically distributed to various tissues where it is further converted intracellularly into the active EIDD-1931 5′-triphosphate moiety by host cell kinases (27). We evaluated the antiviral activity of molnupiravir by studying its major systemic circulating metabolite, EIDD-1931, against a clinical isolate of SARS-CoV-2 (USA-WA1/2020) on A549 cells expressing high levels of angiotensin-converting enzyme 2 (ACE2). Antiviral activity was compared to that of remdesivir, an approved nucleotide analog for COVID-19, and its major systemic circulating metabolite GS-441524 (28). Reduction in infectious viral burden served as the virological endpoint for our assays.

The EIDD-1931 metabolite was more potent against SARS-CoV-2 on ACE2-A549 cells than molnupiravir. The effective concentration associated with 50% of maximum effect (EC_50_) for EIDD-1931 was 11.5-fold lower than that reported for the molnupiravir prodrug, resulting in values of 0.146 μg/mL and 1.617 μg/mL, respectively. EC_95_ concentrations were even more dissimilar with EIDD-1931 yielding a value of 0.324 μg/mL compared to 11.651 μg/mL for molnupiravir, a difference of 36-fold. In contrast, remdesivir was more effective against SARS-CoV-2 than its major circulating metabolite GS-441524. The EC_50_ values for remdesivir were 4.3-fold lower than those reported for GS-441524 (0.218 μg/mL versus 0.927 μg/mL) and EC_95_ values were 3.8-fold lower (0.356 μg/mL versus 1.336 μg/mL). It is important to note that the EC_50_ and EC_95_ values for EIDD-1931 were comparable to the values observed for remdesivir and were markedly lower than those for GS-441524. These findings illustrated the clinical potential of molnupiravir, given that its major circulating metabolite EIDD-1931 was more potent than the major circulating metabolite of remdesivir, a drug that was already approved for the treatment of COVID-19.

### Impact of therapy delay on the antiviral activity of EIDD-1931.

**FIG 1:**
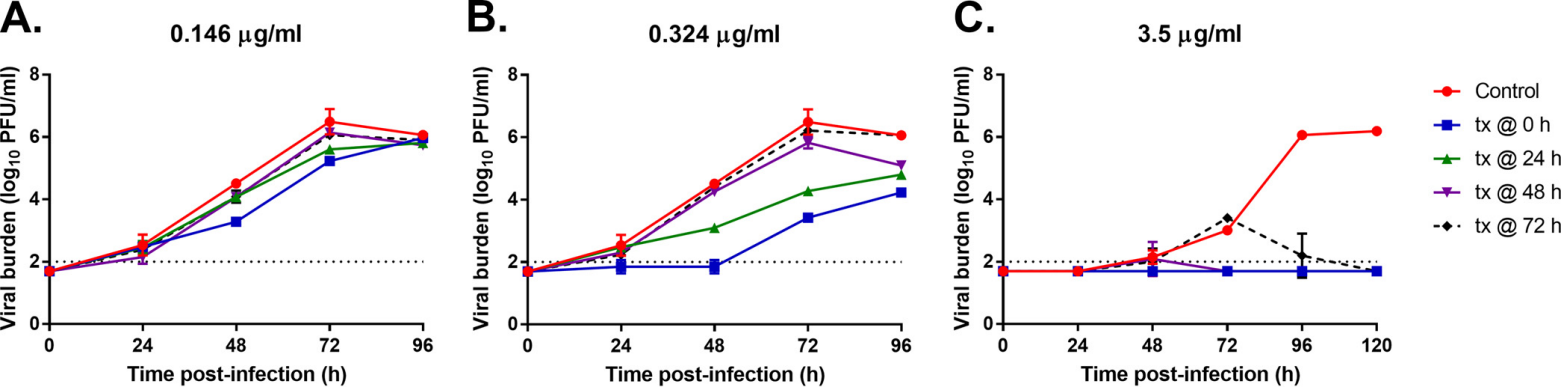
Evaluating the influence of therapy initiation on EIDD-1931 antiviral activity. A549 cells that stably expressed ACE2 were mixed with SARS-CoV-2 (MOI = 10 to 5) and inoculated into the hollow fiber infection model (HFIM). EIDD-1931 therapy (tx) was administered into hollow fiber (HF) cartridges at concentrations equivalent to 0.146 μg/mL (A), 0.324 μg/mL (B), and 3.5 μg/mL (C) beginning at 0 h (tx at 0 h), 24 h (tx at 24 h), 48 h (tx at 48 h), or 72 h (tx at 72 h) postinfection. HF cartridges were sampled daily, and the infectious viral burden was quantified by plaque assay on Vero E6 cells. Data points are reported in log_10_ PFU per mL and represent the mean of two biological replicates ± one standard deviation. The dashed line corresponds to the plaque assay limit of detection (2 log_10_ PFU/mL). Two independent studies were conducted.

We evaluated the influence of delayed therapy initiation on antiviral activity for EIDD-1931 in the hollow fiber infection model (HFIM) inoculated with ACE2-A549 cells and SARS-CoV-2. The major circulating metabolite in the bloodstream (EIDD-1931) was studied instead of the molnupiravir prodrug. EIDD-1931 was administered into the HFIM as a continuous infusion at exposures equivalent to the EC_50_ (0.146 μg/mL) and EC_95_ (0.324 μg/mL) concentrations. Therapy was initiated at 0 h (immediately) or delayed by 24 h, 48 h, or 72 h after infection for each exposure. Antiviral activity directly correlated with time to therapy initiation, as immediate therapy administration provided the greatest amount of viral suppression followed by a 24 h delay for both EC_50_ and EC_95_ (Fig. 1A and B). The impact of time to therapy initiation was more obvious at the EC_95_ exposure because the EC_50_ exposure was only able to delay viral replication for 72 h in the 0 h and 24 h delay arms. By 96 h postinfection, the viral burden was the same in all experimental arms (Fig. 1A). Immediate therapy administration at the EC_95_ exposure resulted in a 3 log_10_ plaque forming unit (PFU)/mL decline in infectious viral burden relative to the control (Fig. 1B) at 72 h postinfection whereas a 24 h delay yielded a 2.2 log_10_ PFU/mL decrease after 3 days of infection. By 96 h, target cell limitation set in from cell death via viral infection, resulting in the expected decline in viral burden in the control (Fig. 1A and B). A therapy administration delay of 48 h and 72 h completely abrogated the antiviral effect. (Fig. 1A and B). This scenario of therapy initiation late in the infection process may reflect the situation in hospitalized patients.

**FIG 2:**
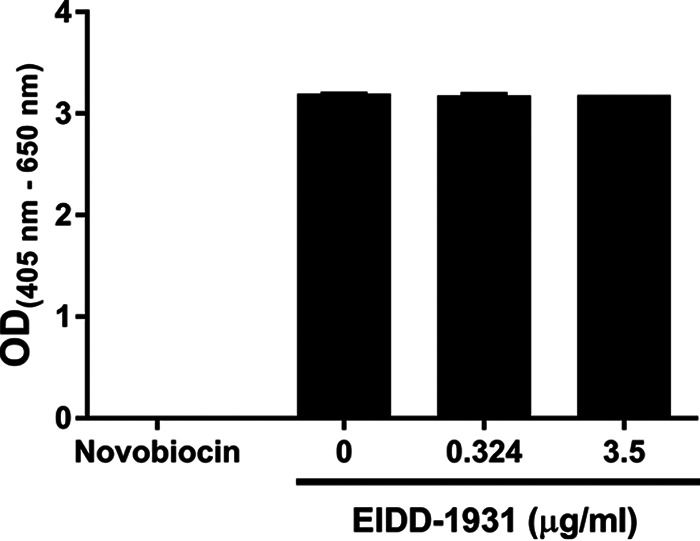
Cytotoxicity of EIDD-1931 on A549 cells that stably express ACE2. ACE2-expressing A549 cells were seeded into 96-well plates at a concentration of 5,000 cells per well. EIDD-1931 was added to cells 24 h after plating and incubated for an additional 72 h. Cell health and metabolism were measured using a WST-1 assay. A 1 mg/mL concentration of novobiocin was used as a positive control for cytotoxicity and cells that did not receive EIDD-1931 served as a negative control. Bars correspond to the mean ± one standard deviation of six biological replicates. Three independent assays were conducted.

We hypothesized that the adverse effects on antiviral activity for EIDD-1931 resulting from therapy initiation delay can be overcome by increasing drug exposure. To examine this hypothesis, we repeated the above-described experiment in the HFIM and administered EIDD-1931 at a concentration equivalent to ~10× EC_95_ (3.5 μg/mL). Viral replication kinetics in the control arm were slower (Fig. 1C) in this experiment compared to the previous experiment (Fig. 1A and B). For that reason, we conducted this high-concentration experiment over 120 h. The higher EIDD-1931 exposure completely suppressed viral replication in all experimental arms by the end of the study (Fig. 1C). Of great interest, the 72-h therapy delay reduced the 3 log_10_ PFU/mL viral burden at the start of therapy down to undetectable levels by 120 h postinfection (after 48 h of therapy). Due to the decline in viral burden, which may be a signal for drug-induced cellular toxicity, we evaluated cell health and metabolism over 72 h under the pressure of 3.5 μg/mL of EIDD-1931 via a water-soluble tetrazolium salt (WST-1) assay. Cell health and metabolism in the drug-exposed arms were not different from untreated controls (Fig. 2), indicating that cellular toxicity was not a factor and that the reduction in viral burden was solely due to the antiviral effect. Thus, higher exposures of EIDD-1931 could overcome the negative influence of delayed therapy initiation and restore antiviral activity, as 3.5 μg/mL was highly effective even after a 72-h delay in therapy initiation.

### Effect of delayed therapy for EIDD-1931 at clinical dosing regimens.

The experiments illustrated in Fig. 1 were all conducted under static drug conditions (continuous infusion). In humans, drug concentrations are generally not static following administration, but are instead dynamic due to absorption, distribution, metabolism, and excretion. Therefore, we aimed to evaluate the effect of delayed therapy initiation using dynamic drug concentration-time profiles and determine if an alternative dosing interval could negate the impact of therapy delay on the antiviral effect of EIDD-1931 against SARS-CoV-2. Pharmacokinetic (PK) profiles (27) associated with 800 mg of EIDD-1931 every 24 h (Q24h) and 400 mg every 12 h (Q12h) were simulated in the HFIM. We accounted for the binding of EIDD-1931 to human plasma proteins, which we identified as 7.8% protein bound by ultrafiltration and assayed by ultraperformance liquid chromatography-tandem mass spectrometry (UPLC-MS/MS). Only free-drug PK profiles were simulated. The static concentration associated with the simulated 24 h area under the concentration-time curve (AUC_0-24h_) exposure in these experiments is 0.324 μg/mL (7.78 mg h/L/24 h = 0.324 μg/mL), which is equivalent to the EC_95_ concentration. EIDD-1931 therapy was delayed by 24 h and 48 h postinfection in the HFIM for both dosage regimens. A high-dose continuous infusion of 3.5 μg/mL was included as a positive assay control.

**FIG 3:**
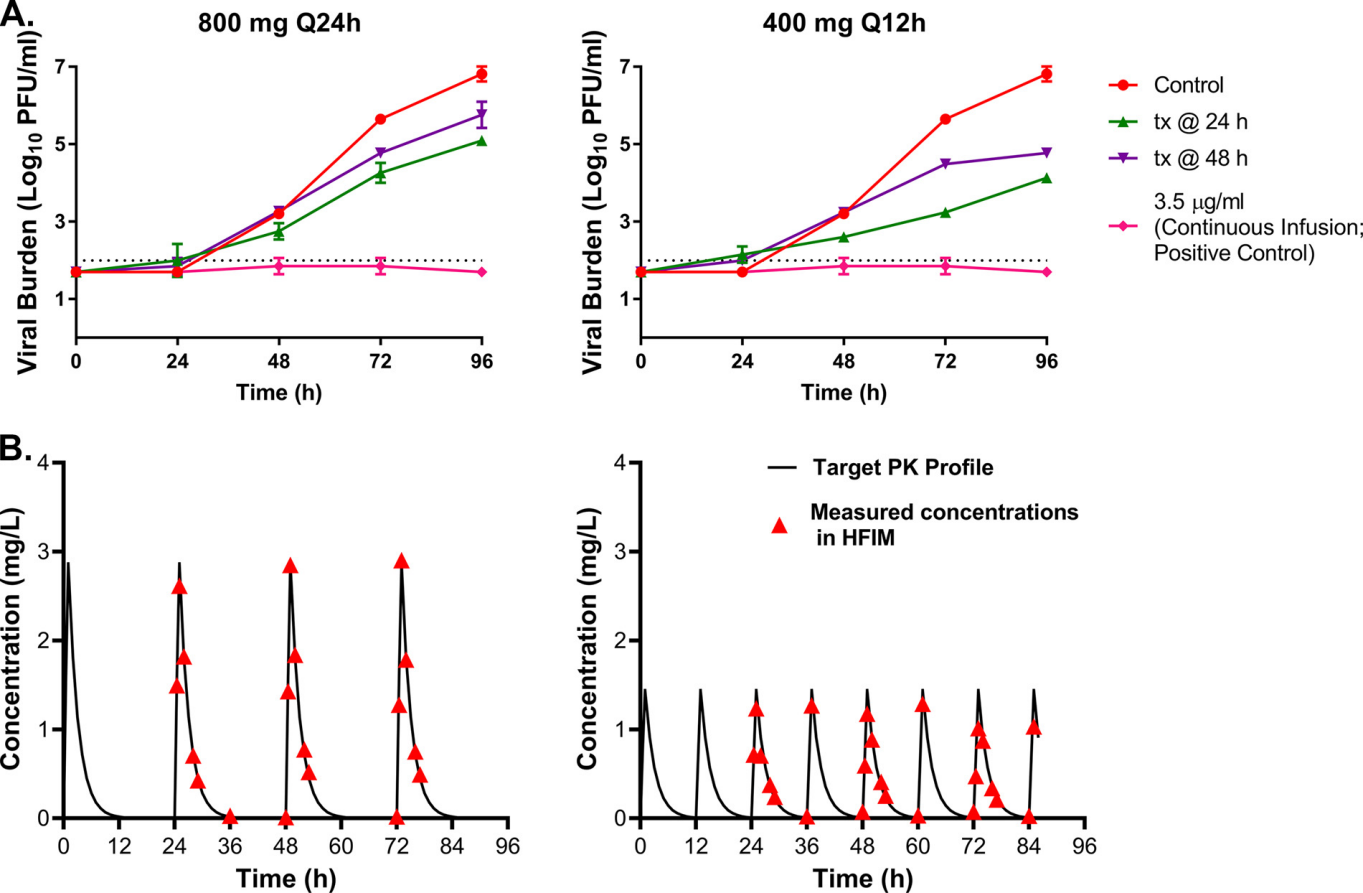
Influence of therapy delay when human pharmacokinetic profiles were simulated for EIDD-1931. (A) A549 cells that stably expressed ACE2 were mixed with SARS-CoV-2 (MOI = 10 to 5) and inoculated into the hollow fiber infection model (HFIM). EIDD-1931 was administered into hollow fiber (HF) cartridges to mimic human pharmacokinetic profiles associated with 800 mg once daily (Q24h) or 400 mg twice daily (Q12h). Therapy was initiated 24 h (tx at 24 h) or 48 h (tx at 48 h) after infection for both dosage regimens. One HF cartridge did not receive the drug, serving as a no-treatment control, and one cartridge received a high dose (3.5 μg/mL) of EIDD-1931 as a continuous infusion served as a positive control. Cartridges were sampled daily, and the infectious viral burden was measured by plaque assay on Vero E6 cells. Data points correspond to the mean ± one standard deviation of two biological replicates and the dashed line signifies the plaque assay limit of detection. Two independent studies were conducted. (B) Medium samples from the central reservoir of each HFIM system were taken multiple times throughout the first 48 h to ensure the desired PK profiles were achieved. Concentrations of EIDD-1931 were quantified via liquid chromatography-tandem mass spectrometry.

As expected, the earlier therapeutic intervention resulted in greater viral suppression when dynamic drug-concentration profiles were simulated (Fig. 3A and B). With Q24h administration, a delay of 24 h provided an extra 0.5 log_10_ PFU/mL of viral suppression relative to a 48-h delay (Fig. 3A). The influence of timing of therapy initiation was more pronounced with Q12h dosing, as a 24 h delay reduced viral burden by 1.25 log_10_ PFU/mL more than the experimental arm that received EIDD-1931 48 h after infection (Fig. 3B), indicating that Q12h dosing resulted in greater viral suppression compared to the corresponding Q24h dosing arm when EIDD-1931 was delivered as a dynamic infusion (Fig. 3). The area under the viral burden-time curve (AUC_VB-t_) was 298 log_10_ PFU/mL/h (95% confidence interval [CI] of 279.8 to 316 log_10_ PFU/mL/h) for the Q24h dosing 24 h after infection compared to 261.7 log_10_ PFU/mL/h [95% CI, 254.1 to 269.4 log_10_ PFU/mL/h] for the Q12h dosing with a 24 h delay. This finding suggested that more frequent dosing intervals were required to maximize inhibition of viral replication and that a percentage time > EC_95_ (Q24h = 23.5% and Q12h = 33.8%) was the pharmacodynamic (PD) index best linked to the antiviral effect for EIDD-1931 (molnupiravir). However, as the delay of therapy became longer (48 h), the differences between the Q24h and Q12h dosing were smaller (AUC_VB-t_, Q24h = 326.8 log_10_ PFU/mL/h [95% CI, 315.6 to 338.1] versus Q12h = 311.3 log_10_ PFU/mL/h [95% CI, 308.8 to 313.8]), illustrating a loss of antiviral potency with later stage therapy initiation. This directly demonstrated that more frequent dosing intervals alone could not negate the adverse influence of therapy delay on antiviral activity. The viral burden was at or below the limit of detection in the positive control, illustrating that replication kinetics could be completely shut down when enough drug was present (Fig. 3A and B).

EIDD-1931 concentrations were measured in the HFIM by UPLC-MS/MS to ensure that the desired concentration-time profiles were achieved (Fig. 3C). All measured concentrations were within 10% of the targeted value, demonstrating that the appropriate PK profiles were attained in the HFIM.

### Mechanism for loss of antiviral activity with therapy initiation delay.

**FIG 4:**
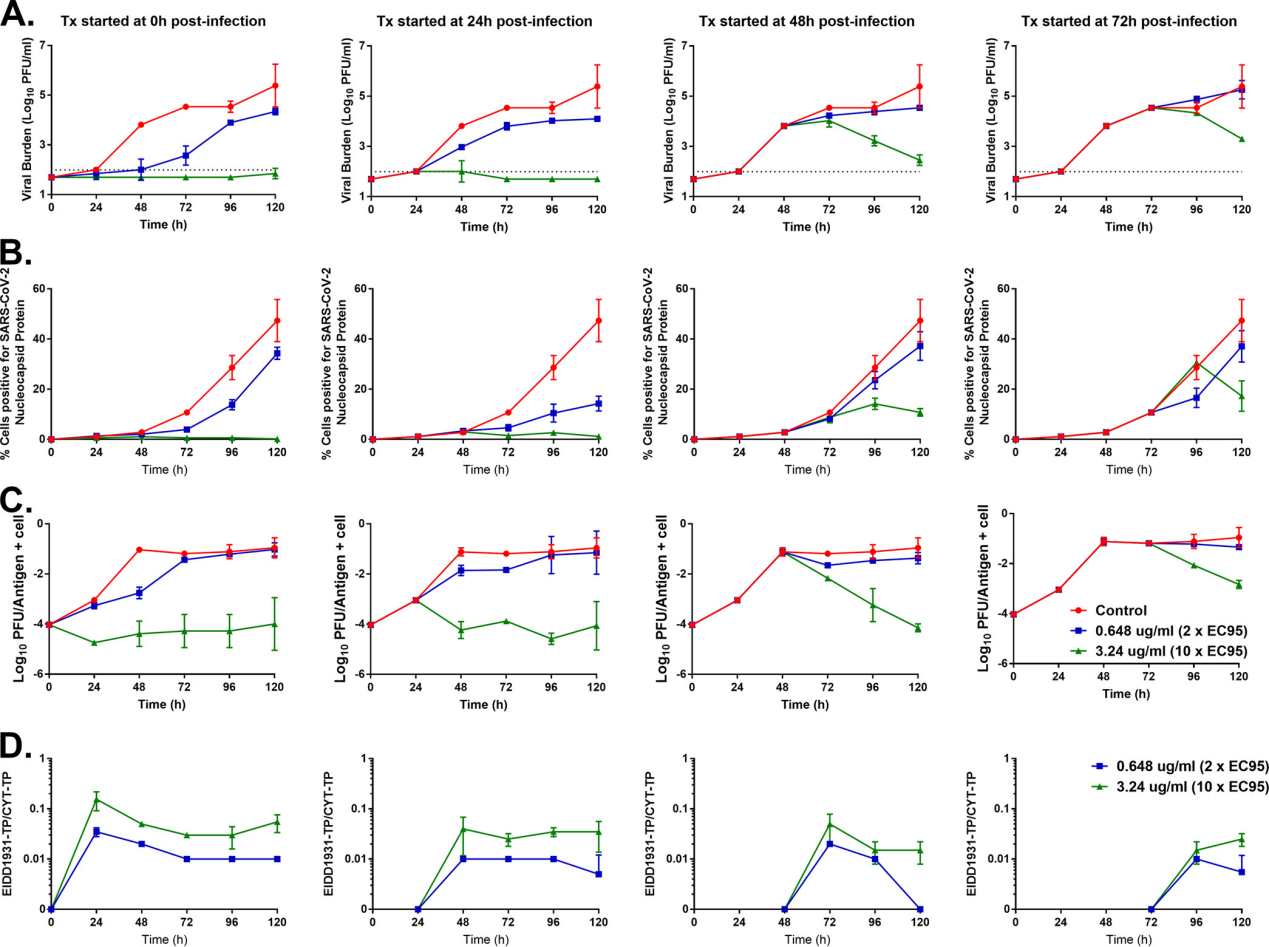
The effect of therapy initiation delays on intracellular viral production and spread as well as EIDD-1931 metabolism. Tissue culture flasks seeded with ACE2 expressing A549 cells were infected with SARS-CoV-2 at an MOI = 0.03. EIDD-1931 was added to cell monolayers at concentrations equivalent to 0.648 μg/mL and 3.24 μg/mL at 0 h, 24 h, 48 h, or 72 h postinfection. Control flasks that did not receive drugs served as a negative control. Viral supernatants and cells were harvested daily beginning 24 h posttherapy initiation for up to 120 h postinfection. (A) The concentration of extracellular infectious viral burden was determined by performing plaque assays on viral supernatant samples. (B) Flow cytometry was performed on a portion of harvested cells to determine the amount of cell-to-cell viral spread over time. Cells were fixed and stained with an antibody specific to the SARS-CoV-2 nucleocapsid (NP) protein. (C) The intracellular infectious viral burden was determined by performing a plaque assay on a portion of the harvested cells. The intracellular viral burden is reported as log_10_ PFU per antigen (NP) positive cells. (D) The ratio of EIDD-1931 triphosphate (TP) to CTP (CYT-TP) was determined from the remaining harvested cells. Cells were inactivated with a mixture of acetonitrile and methanol for 30 min and frozen at −80°C. Intracellular levels of EIDD-1931-TP and CYT-TP were determined via ultraperformance liquid chromatography-tandem mass spectrometry. All data points represent the mean of two biological replicates and two independent studies were conducted.

We sought to explain the negative impact of therapy initiation delay by identifying the mechanism that resulted in the loss of antiviral activity for EIDD-1931 against SARS-CoV-2 at later stages of infection. We hypothesized that viral replication over time as well as the ratio of triphosphate pools for EIDD-1931 and cytidine (the endogenous host cell nucleotide that EIDD-1931 mimics) were the driving factor behind this phenomenon. The ACE2-A549 cell line was adherent and, consequently, we could not serially sample cells from the HFIM system because the cells attached to the fibers. Therefore, we performed an additional experiment in tissue culture flasks to evaluate extracellular infectious viral burden, cell-to-cell viral spread, intracellular infectious viral burden, as well as EIDD-1931-triphosphate and cytidine-triphosphate concentrations in SARS-CoV-2 infected ACE2-A549 cells over time at extracellular EIDD-1931 concentrations of 0.648 μg/mL (equivalent to 2× EC_95_) and 3.24 μg/mL (equivalent to 10× EC_95_). The drug was administered immediately (0 h), as well as 24 h, 48 h, and 72 h after infection (Fig. 4).

Unsurprisingly, earlier therapy initiation resulted in greater viral suppression for the extracellular infectious virus (Fig. 4A). EIDD-1931 at 0.648 μg/mL delayed viral replication when administered at 0 h and 24 h postinfection, and the degree of suppression correlated with time to therapy initiation. For example, at 72 h postinfection infectious viral burden was reduced by 2 log_10_ PFU/mL when the drug was immediately administered (72 h of drug exposure) whereas a reduction of only 0.74 log_10_ PFU/mL was observed when therapy was delayed by 24 h (i.e., after 48 h of drug exposure) at the same time point (Fig. 4A). Antiviral activity was completely abrogated at this exposure of EIDD-1931 when therapy was delayed by 48 h and 72 h postinfection. The higher drug concentration (3.24 μg/mL) completely suppressed viral production when therapy was initiated early because the viral burden was at or below the assay limit of detection for the entire study at the 0 h and 24 h therapy initiation experimental arms (Fig. 4A). EIDD-1931 at 3.24 μg/mL was also effective when administered later after infection and resulted in a steady decline in infectious viral titers even after a 72-h delay in therapy initiation. These findings further demonstrated that the attenuated antiviral effect due to delayed therapy initiation could be partially negated by higher drug exposures.

EIDD-1931 slowed down cell-to-cell viral spread at 0.648 μg/mL and nearly inhibited spread at the higher 3.5 μg/mL concentration when therapy was administered at 0 h or 24 h postinfection as determined by flow cytometry (Fig. 4B). When therapy was initiated at 48 h and 72 h postinfection, 0.648 μg/mL failed to suppress viral spread because the percentage of cells positive for the SARS-CoV-2 nucleocapsid protein in these experimental arms were nearly identical to the control (Fig. 4B). EIDD-1931 at 3.24 μg/mL was able to reduce the amount of viral spread between cells when administered 48 h after infection but was ineffective when therapy was delayed by 72 h.

Intracellular infectious viral burden closely mimicked the trends observed for the extracellular viral burden profiles discussed above (Fig. 4A and C), but replication kinetics of intracellular virus was slightly faster than those exhibited by the extracellular virus (Fig. 4C). Earlier administration of EIDD-1931 resulted in greater levels of viral suppression, with 0.648 μg/mL delaying viral replication and 3.24 μg/mL suppressing replication (Fig. 4C) in the 0 h and 24 h therapy initiation experimental arms. The lower concentration of the drug failed to inhibit the infectious virus intracellularly when therapy initiation was delayed by 48 h and 72 h postinfection. The higher concentration of the drug, however, caused a viral burden to decline steadily over time, driving down the viral burden through 120 h postinfection even when therapy was initiated 72 h after infection (Fig. 4C).

Finally, we measured the intracellular concentrations of EIDD-1931-triphosphate and cytidine-triphosphate in the infected ACE2-A549 cells over time following exposure to 0.648 μg/mL and 3.24 μg/mL of EIDD-1931 (Fig. 4D). The ratio of EIDD-1931-triphosphate to cytidine-triphosphate was compared between the different drug concentrations over time for each regimen. The addition of EIDD-1931 to cells immediately following SARS-CoV-2 infection resulted in an initial peak in EIDD-1931-triphosphate at 24 h after therapy before plateauing at a ratio of approximately ~0.01 and ~0.04 for the 0.648 μg/mL and 3.24 μg/mL concentrations, respectively (Fig. 4D). Triphosphate ratios remained stable throughout the experiment in these samples. Similar findings were observed between the two EIDD-1931 concentrations when therapy was delayed by 24 h, except for the initial spike in EIDD-1931-triphosphate (Fig. 4D). A 2-fold decrease in this ratio, signifying a decrease in EIDD-1931 triphosphate pools, was seen for the 3.24 μg/mL concentration when therapy was delayed by 48 h and 72 h. Interestingly, triphosphate pools were relatively unchanged with delay in therapy at the lower drug concentration which retained ratios of ~0.01 throughout the study except for a single outlier point at 120 h in the 48 h therapy delay experimental arm.

## DISCUSSION

Antiviral agents against SARS-CoV-2 are critical to mitigate the ongoing COVID-19 pandemic and there has been a concentrated push to identify potent drugs to treat patients infected with this virus. This concerted effort has led to the development and now clinical use of multiple drugs for the treatment of COVID-19 (22, 25, 29). However, effective antiviral intervention is still lacking for hospitalized patients with more severe disease (19, 26). Molnupiravir is an oral nucleoside analog that is efficacious in an outpatient setting (25) but was shown to be ineffective in patients hospitalized for SARS-CoV-2 infection (26). Thus, molnupiravir is currently only available under emergency use authorization for the treatment of adults with mild-to-moderate COVID-19 (30).

The importance of early treatment initiation for antivirals has been well documented for other acute viral illnesses such as influenza. Clinical trials have shown that neuraminidase inhibitors (oseltamivir and zanamivir) are effective when administered 48 h after the onset of symptoms (31, 32). However, oseltamivir retains some modest efficacy, albeit reduced, in children up to 3 days postsymptom onset, suggesting that therapy initiation outside the recommended two-day window may be beneficial for oseltamivir (33). Our findings showed that a similar approach should be leveraged for antiviral administration for SARS-CoV-2, and therapy should be administered as soon as possible to maximize viral suppression and improve therapeutic outcomes in patients with COVID-19.

Nucleoside/nucleotide analogs comprise a class of potent antiviral drugs that have a long and successful history as a therapeutic strategy against several important viral diseases, including HIV and HCV (34–37). Therefore, it is perplexing why molnupiravir did not improve clinical outcomes in hospitalized patients with COVID-19 (26). Here, we aimed to explain why molnupiravir failed in patients with severe disease but not a mild-to-moderate disease and determine if larger doses and optimized dosing intervals could overcome treatment failures. We hypothesized that the overall delay in treatment initiation in hospitalized patients was to blame for the lack of efficacy. It is important to note that this hypothesis was made before the completion and publication of the MOVe-IN and MOVe-OUT trials. We employed static tissue culture systems and the dynamic HFIM for SARS-CoV-2 to test our hypothesis. Because molnupiravir is rapidly converted to EIDD-1931 in human plasma following oral administration (27), our *in vitro* studies used EIDD-1931 in place of the molnupiravir prodrug for all the antiviral evaluations.

Our results demonstrated that the delay of therapy initiation markedly decreased the antiviral effectiveness of EIDD-1931 and the degree of effectiveness declined as the time to therapy administration and the infection gets longer (Fig. 1 and 3). These findings were consistently obtained in the HFIM regardless of the schedule of administration because attenuated antiviral effectiveness was observed when EIDD-1931 was administered as a continuous infusion or as a dynamic infusion to mimic human PK concentrations associated with clinical dosage regimens. These data suggested that shorter dosing intervals were unlikely to overcome the negative influence of therapy delay on antiviral activity for molnupiravir without increasing the overall dose and thus drug exposure.

We also showed that substantially higher exposures to a drug (10× EC_95_) can restore the antiviral activity of EIDD-1931 when administered later in the viral infection process and, surprisingly, reduce infectious viral burden over time. It is important to note how much more drug was required to overcome the deleterious effects of delay of therapy onset because 2× EC_95_ concentrations were not effective when administered later in infection (Fig. 4D). The AUC_24h_ exposure associated with the current clinical molnupiravir dosage regimen (800 mg twice daily) (27) was equivalent to a static concentration of 0.648 μg/mL or 2× EC_95_. Thus, simulating this regimen in our HFIM studies (Fig. 2) would be unlikely to change the results and our subsequent interpretation.

The reduction in viral burden resulting from therapy with high concentrations of EIDD-1931 is likely attributable to the mechanism of action of molnupiravir, which serves as a lethal viral mutagen (24). Thus, cells exposed to EIDD-1931 produce viral particles that contain mutated viral RNA that is incapable of further rounds of replication. We hypothesize that these viral particles act as defective interfering particles, competing with infectious viral particles for the same host cell receptor. The increase and release of mutated viral particles will drive down the infectivity of viral supernatants, as defective viruses outcompete infectious viruses for binding and uptake into the cell. This phenomenon has been well described for coronavirus and influenza virus in the absence of drug therapy (38, 39).

It is important to understand why therapy delay has a negative impact on antiviral activity. We looked inside the cell to identify the mechanism behind this phenomenon beginning with the quantification of the intracellular EIDD-1931 triphosphate pool (Table S1). Higher concentrations of extracellular EIDD-1931 resulted in the formation of more EIDD-1931-triphosphate (Table S1). These observations suggested that if the phosphorylation was a saturable (i.e., Michaelis-Menten) process, the saturation would only occur at higher drug concentrations. Thus, the rate of EIDD-1931-triphosphate formation was near linear at our studied EIDD-1931 concentrations. We also showed that the ratio of intracellular EIDD-1931-triphosphate to cytidine-triphosphate (the natural substrate) was stable over time, particularly when the drug was administered early after infection (Fig. 4D). The information regarding phosphorylation kinetics of EIDD-1931 described here can be further modeled to link EIDD-1931/EIDD-1931 triphosphate concentrations to effect.

10.1128/mbio.02916-22.2 TABLE S1 Intracellular concentrations of EIDD-1931 in SARS-CoV-2-infected ACE2-expressing A549 cells. Download Table S1, DOCX file, 0.01 MB. Copyright © 2022 Brown et al. 2022 Brown et al. https://creativecommons.org/licenses/by/4.0/ This content is distributed under the terms of the Creative Commons Attribution 4.0 International license.

In addition to EIDD-1931 metabolism, viral replication was rapid inside the cell (Fig. S1, Text S1) Therefore, the amount of intracellular infectious viral burden and viral RNA is substantially higher at later time points (≥48 h) postinfection. When therapy was initiated early in the infection process the levels of intracellular virus/vRNA were low. Conversely, when therapy was delayed and initiated later postinfection the levels of intracellular virus/vRNA were high. EIDD-1931 triphosphate was competing with CTP for uptake by the viral RNA-dependent RNA polymerase and subsequent incorporation into the nascent viral RNA strand. When intracellular viral levels are low, the number of viral polymerase proteins was also low. Thus, there was a greater chance that the viral polymerase would bind to and incorporate available EIDD-1931 triphosphate into replicating viral RNA genomes. With lower levels of vRNA at the start of therapy, a high fraction of intracellular vRNA will likely acquire enough of the drug to result in lethal mutagenesis. Most viral particles released from EIDD-1931 exposed cells will be replication-defective and unable to propagate the infection, resulting in an antiviral effect.

10.1128/mbio.02916-22.1 FIG S1 Levels of intracellular viral RNA increase substantially over time. Tissue culture flasks seeded with ACE2 expressing A549 cells were infected with SARS-CoV-2 at an MOI = 0.03. An uninfected control flask was included as a negative control. Cells were harvested daily beginning at 0 h posttherapy initiation for up to 72 h. RNA was extracted from infected cells and RNA was quantified via a quantitative real-time reverse transcriptase polymerase chain reaction assay. The increase of viral RNA was calculated relative to the 0 h time point using the 2^-Δct^ method. Download FIG S1, TIF file, 0.1 MB. Copyright © 2022 Brown et al. 2022 Brown et al. https://creativecommons.org/licenses/by/4.0/ This content is distributed under the terms of the Creative Commons Attribution 4.0 International license.

10.1128/mbio.02916-22.3 TEXT S1 Supplemental methods for quantifying intracellular levels of viral RNA. Download Text S1, DOCX file, 0.01 MB. Copyright © 2022 Brown et al. 2022 Brown et al. https://creativecommons.org/licenses/by/4.0/ This content is distributed under the terms of the Creative Commons Attribution 4.0 International license.

Conversely, a higher viral burden later in infection resulted in a larger amount of VRNA and viral polymerase proteins that were competing for the fixed amount of EIDD-1931 triphosphate (because levels did not radically change over time). The probability of drug incorporation by the viral polymerase was substantially decreased in this scenario because the increase in vRNA (and polymerase proteins) diluted out the amount of active drug available for the replicating viruses. Consequently, only a fraction of the nascent vRNA strands contained enough EIDD-1931 triphosphate to result in lethal mutagenesis. The remaining vRNA was replication-competent and packaged and released from the cell to productively infect a new host cell target, thereby propagating the infection and resulting in a decrease in antiviral activity.

Higher exposures of EIDD-1931 were able to overcome the negative effects of therapy delay due to the increased levels of EIDD-1931 triphosphate that these concentrations provided. The addition of drug triphosphate served to reduce the dilution effect resulting from a high intracellular viral load at the start of therapy by increasing the likelihood of drug incorporation into a higher proportion of newly replicating vRNA genomes, restoring the antiviral effect. It has been reported that patients with severe COVID-19 tend to display a higher viral burden than those with mild-to-moderate disease (40). In the context of our findings, it is possible that increasing the intensity of molnupiravir therapy may be an effective strategy to improve therapeutic outcomes in hospitalized patients. Our studies suggested that 5-times the current clinical dosage regimen may be required to achieve this robust antiviral effect in these very sick patients. This exposure may not be tolerated. Alternative dosing strategies, including molnupiravir as a combination therapy with agents from different drug classes, should be investigated as a therapeutic strategy to overcome exposure-related limitations in hospitalized patients.

There are several limitations to our study. First, our experiments were conducted using only the originating strain of SARS-CoV-2. Although current data suggest that molnupiravir effectiveness is not altered against more contemporary SARS-CoV-2 variants, we will evaluate the activity of EIDD-1931 against clinically relevant viral isolates (e.g., Omicron). A second limitation is that antiviral evaluations were only conducted in ACE2-A549 cell lines. While cell lines are a useful tool for preclinical antiviral studies, activity may change when primary human cells are employed. We plan to utilize primary human airway epithelial cells and primary human lung cells to validate our findings. Finally, it is important to note that our experimental evaluations were performed in the absence of an immune system. The presence of an immune component in addition to EIDD-1931 would likely provide additional viral suppression. However, the immune system is also the cause of the inflammatory response (e.g., “cytokine storm”) during COVID-19, which contributes to disease. Thus, treating hospitalized patients later in the disease process is likely more complicated than just controlling viral replication through antiviral therapy and may need to include strategies to control the immune response.

**FIG 5:**
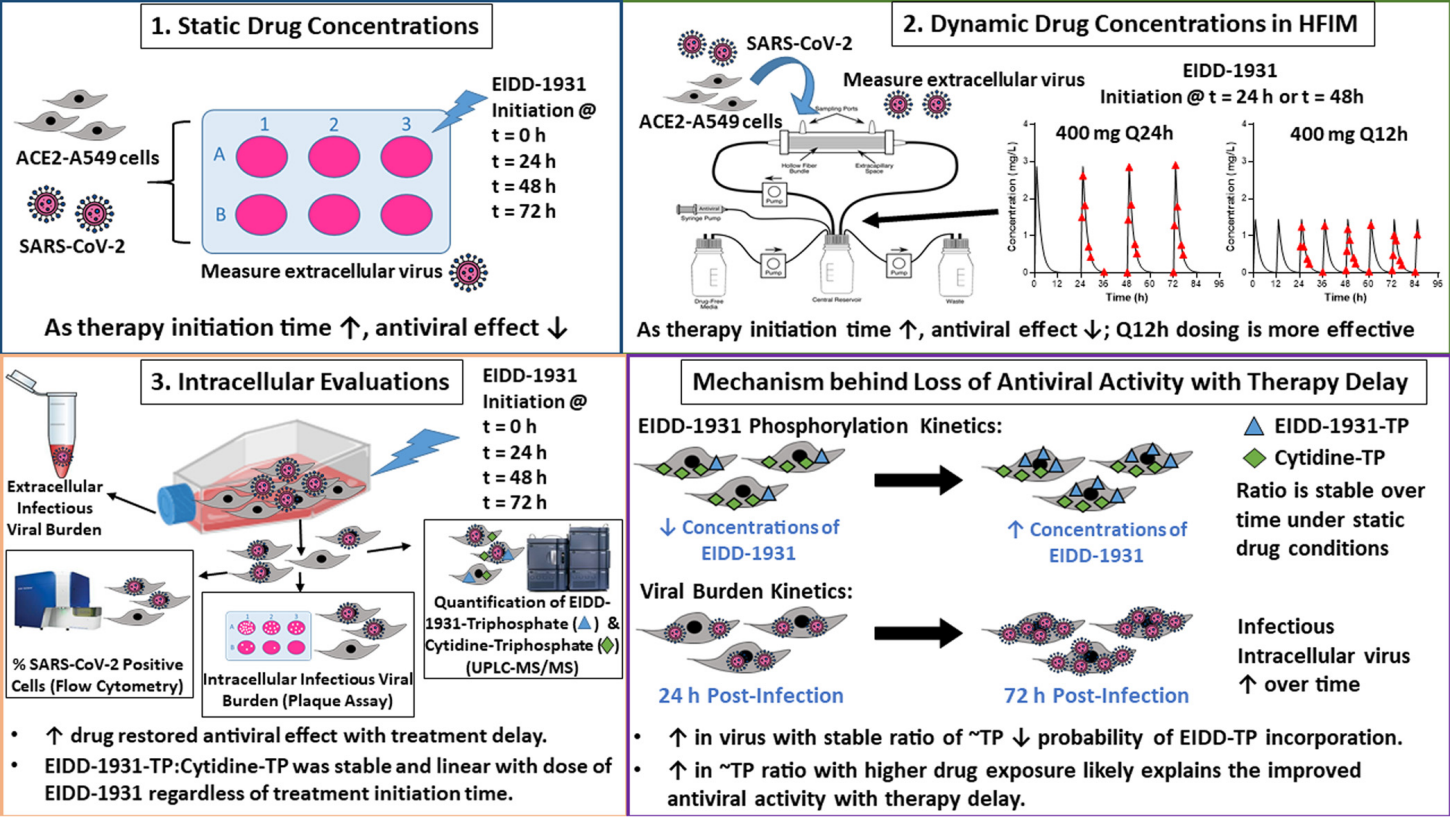
Summary of key experimental findings. This illustration summarizes the key experiments and the corresponding findings conducted in this study. The outline of our overall mechanism explaining the loss of antiviral activity with the delay of therapy initiation is shown in the bottom right panel.

Here, we explained why molnupiravir failed for the treatment of COVID-19 in hospitalized patients, due to the late therapy initiation (Fig. 5). The studies described were conducted before the termination and publication of the MOVe-IN and MOVe-OUT trials. From our data and analyses, we correctly (and prospectively) predicted the failure of the MOVe-IN trial as well as the positive outcome associated with the MOVe-OUT trial. Our findings illustrated that molnupiravir was most effective at combatting COVID-19 when administered as early as possible postinfection. Currently available COVID-19 vaccines are unable to prevent SARS-CoV-2 transmission (41, 42). Given the EIDD-1931 potency demonstrated here with early therapy initiation, it would be wise to consider administering molnupiravir as postexposure prophylaxis in people exposed to SARS-CoV-2. Drug administration before or at the beginning of symptom onset may be the best way to prevent further transmission of the virus, thereby breaking the back of this pandemic. We propose that molnupiravir as a combination therapy may be one strategy to increase drug exposure with limited toxicity to effectively treat patients with severe COVID-19. Importantly, this experimental approach may also be applied to other antiviral agents active against COVID-19 or other viral diseases; and, in addition to evaluating antiviral activity, these *in vitro* model systems can also be employed to identify optimized dosing strategies to prevent the emergence of drug-resistance to maintain the long-term clinical utility of these agents. These are currently active areas of research in our laboratory.

## MATERIALS AND METHODS

### Cells, viruses, and compounds.

Vero E6 cells were purchased from the American Type Culture Collection (ATCC CRL-1586) and ACE2 transfected A549 cells (ACE2-A549 cells) (43) were a kind gift from Shinji Makino at the University of Texas Medical Branch. Both cell lines were maintained as previously described (44, 45) and subcultured twice weekly to ensure subconfluency. The USA-WA1/2020 SARS-CoV-2 strain was obtained through Biodefense and Emerging Infectious Research Resources Repository (BEI Resources, NR-52281), and viral stocks were propagated on Vero E6 cells (45). All virus experiments were conducted in a biosafety level 3 facility using approved protocols. Molnupiravir, EIDD-1931, remdesivir, and GS-441524 were all purchased from Medkoo Biosciences. Cytidine, CTP (CYT-TP), isotope-labeled (^13^C_5_) cytidine, and isotope-labeled (^13^C_5_) CYT-TP were purchased from Toronto Research Chemicals. Drug powder was reconstituted in 100% DMSO for all antiviral experiments.

### Antiviral evaluations in tissue culture plates.

Antiviral assays employing static drug concentrations for molnupiravir, EIDD-1931, remdesivir, and GS-441524 were conducted in 6-well plates with ACE2-A549 cells and performed as previously described (44). Five drug concentrations were evaluated per compound along with a no-treatment control. Viral supernatants were sampled daily for 4 days. Supernatants were clarified by high-speed centrifugation and frozen at −80°C until the end of the study. The infectious viral burden was determined simultaneously for all samples by plaque assay on Vero E6 cells (44, 45). EC_50/95_ values were calculated over the entire four-day experiment as described previously (44) using GraphPad Prism software version 7.02 (GraphPad Software).

### Antiviral evaluations in the HFIM using static drug concentrations.

Antiviral evaluations for EIDD-1931 were conducted in the HFIM to determine the effect of delayed therapy initiation on antiviral activity. A description of the HFIM for viral pathogens can be found elsewhere (46, 47). Briefly, 10^8^ ACE2-A549 cells were mixed with 10^3^ PFU of SARS-CoV-2 and inoculated into the extracapillary space of cellulosic hollow fiber (HF) cartridges (FiberCell Systems). EIDD-1931 was administered into HF cartridges as a continuous infusion at concentrations equivalent to 0.146 μg/mL (EC_50_), 0.324 μg/mL (EC_95_), and 3.5 μg/mL (~10× EC_95_) immediately after infection (0 h) or 24 h, 48 h, and 72 h postinfection to simulate delayed therapy initiation. A no-treatment control cartridge was included, for a total of 13 HF cartridges. HF cartridges were sampled daily in duplicate for 4 days. Samples were clarified by high-speed centrifugation and frozen at −80°C until the end of the study. The infectious extracellular virus was determined by plaque assay on Vero E6 cells.

### Antiviral evaluations in the HFIM simulating human PK profiles.

Antiviral evaluations in which EIDD-1931 was administered dynamically to mimic human PK profiles associated with clinical dosage regimens for molnupiravir were conducted in the HFIM. Six HF cartridges were employed for this study. EIDD-1931 was administered into four HF cartridges via computer-controlled syringe pumps as a 1-h infusion to achieve a free-drug area under the 24 h concentration-time curve (AUC_0-24h_) exposure associated with a total daily molnupiravir dose of 800 mg (AUC_0-24h_ = 7.78 mg h/L) (27). We determined that the binding of EIDD-1931 to human plasma proteins was ~7.8%, as discussed below, and only free-drug concentrations were simulated in this study. Two cartridges received the total AUC_0-24h_ exposure once daily (Q24h) and two received half the daily AUC_0-24h_ exposure twice daily (Q12h). Therapy was initiated either 24 h or 4 8 h postinfection for both the Q24h and Q12h regimens. EIDD-1931 was eliminated from the HF systems at a rate to mimic a 1.5 h half-life for each dosage regimen. One cartridge did not receive the drug and served as a no-treatment control, and one served as a positive control in which EIDD-1931 was administered at a high concentration (3.5 μg/mL) as a continuous infusion. Cartridges were serially sampled daily for 4 days and the infectious viral burden was quantified in samples, as described above.

### Bioanalytical methods for pharmacokinetic validation in the HFIM.

During the first 48 h of the HF studies, medium from the central reservoir of each HF system was sampled at various times postdrug infusion to quantify the EIDD-1931 concentration and ensure that the desired PK profiles were achieved. Samples were inactivated to remove any potential viral contamination by incubating 200 μL of the media sample with a 1 mL solution of methanol and acetonitrile (1:1, vol/vol) for 30 min at room temperature. Samples were then removed from the BSL-3 laboratory and frozen at −80°C until quantification by ultraperformance liquid chromatography-tandem mass spectrometry (UPLC-MS/MS).

Calibration standard curves were prepared by spiking 20 μL of serial working standard solutions into 180 μL of blank cell culture medium to achieve final concentrations of 0.03 to 100 μmol/L. One milliliter of methanol/acetonitrile was added to each standard (as described above). Proteins in the methanol/acetonitrile-containing solution were separated by centrifugation at 13,000 rpm for 10 min. The supernatant extract was diluted and injected into an Acquity I-Class UPLC system (Waters) interfaced with a Triple Quad 6500+ MS/MS system (AB Sciex). The UPLC separation of EIDD-1931 was performed using an Atlantis TM Premier BEH C18 AX 100 × 2.1 mm, 1.7 μm column (Waters, Milford, MA) with a run time of 3.5 min. The mobile phase consisted of 20 mM ammonium bicarbonate in water (A) and acetonitrile (B) at a flow rate of 0.3 mL/min using a gradient elution method. The Triple Quad 6500+ MS/MS system was operated in negative ion mode using the turbo spray IonDrive^TM^. The mass transition monitored for quantification was 257.9 to 125.9 *m/z* for EIDD-1931, and the transition of 257.9 to 168.0 *m/z* was additionally monitored as a qualifier. The UPLC-MS/MS peak integration and data analysis were performed in the Analyst software package (AB Sciex, Framingham, MA). Precision was 12.2, 2.9, 8.5, 6.5, and 7.3%, and accuracy was 2.5, −3.7, 10.3, 2.3, and −8.7% at 0.03, 0.1, 0.6, 3, and 30 μmol/L EIDD-1931, respectively, with 0.03 μmol/L representing the lower limit of quantification (LLOQ). Measured drug concentrations were within 10% of the desired profile for all studies in the HFIM. Protein binding analysis of EIDD-1931 in human plasma used the same bio-analytical procedures as described above.

### Cytotoxicity evaluation.

Cytotoxicity related to EIDD-1931 therapy was measured using the commercially available WST-1 cell proliferation assay (Roche Diagnostics GmbH) according to the manufacturer’s instructions and as previously described (44).

### Antiviral studies to determine the mechanism of adverse effects related to therapy delay.

For these studies, ACE2-A549 cells were seeded into 122 T-150cm^2^ flasks and allowed to grow to confluence. Cells were infected with SARS-CoV-2 at a multiplicity of infection (MOI) equal to 0.03, as previously described (44). EIDD-1931 at concentrations of 0.648 μg/mL (2× EC_95_) and 3.24 μg/mL (10× EC_95_) were administered to flasks immediately after infection (0 h) or therapy was delayed by 24 h, 48 h, or 72 h postinfection. No-treatment control flasks were included for each time point of the study. Flasks for each EIDD-1931 concentration (control, 0.648 μg/mL, and 3.24 μg/mL) at each therapy start time (0 h, 24 h, 48 h, and 72 h) were harvested every 24 h posttherapy initiation for up to 120 h. Viral supernatants and cells were harvested at each time point. Viral supernatants were clarified and stored at −80°C until the end of the study. The extracellular infectious viral burden was quantified in these samples by plaque assay on Vero E6 cells. The number of live harvested cells was enumerated using the trypan blue exclusion test and a hemocytometer. Two aliquots containing 2 × 10^6^ live cells were used for flow cytometry to determine the percentage of cells infected with SARS-CoV-2. Cell pellets were suspended in 5% formaldehyde and fixed overnight at 4°C. The following day, cell pellets were washed twice in wash buffer (PBS supplemented with 1% [wt/vol] saponin, 0.9% [wt/vol] sodium azide, and 5 g/L [wt/vol] bovine serum albumin) by centrifugation at 400 × *g* for 5 min. The pellet was then resuspended in a 1:100 dilution of an Alexa Fluor 488 conjugated rabbit anti-SARS-CoV-2 nucleocapsid antibody (Novus Biologicals) in a wash buffer for 1 h at room temperature. Cells were washed two additional times in wash buffer followed by a final wash in PBS. Cells were suspended in 500 μL PBS and analyzed on a BD FACSVerse flow cytometer (BD Biosciences). Live cells were gated based on size and complexity and 10,000 live events were collected. Data were analyzed using BD FACSuite software.

A portion of the cell pellet (~10^7^ cells) was suspended in PBS, pelleted, and frozen at −80°C until the end of the study. The amount of intracellular infectious viral burden was determined by plaque assay on Vero E6 cells. Briefly, cell pellets were thawed and serially diluted 10-fold ranging from concentrations of 10^6^ cells/mL to 10 cells/mL. A 100 μL aliquot of each cell suspension was added to confluent Vero E6 cell monolayers seeded into 6-well plates. Incubation times and overlays were conducted as described for the plaque assay (44, 45). The number of cells used to calculate viral titer was multiplied by the percentage of cells infected, as determined by the flow cytometry assay, to yield the number of antigen-positive cells in that sample. The intracellular infectious viral burden is reported as log_10_ PFU/antigen positive cell.

### Infected cells sample acquisition and preparation for UPLC-MS/MS analysis.

Finally, the remaining cells (~3 × 10^7^ cells) were aliquoted into three separate microcentrifuge tubes, pelleted, resuspended in 2 mL of methanol: acetonitrile (1:1, vol/vol) solution, vortexed for 20 s, and incubated for 30 min at room temperature to inactivate any infectious virus in the sample. Samples were then removed from the BSL-3 laboratory and frozen at −80°C before intracellular levels of EIDD-1931, EIDD-1931 triphosphate (NHC-TP), cytidine and CTP (CYT-TP) were quantified by UPLC-MS/MS.

Samples were prepared by serial steps of breaking cells, concentrating, resuspension, and protein precipitation. Briefly, the methanol and acetonitrile were removed by a 24-position MICROVAP nitrogen evaporator after the cells were lysed. The dry cell lysate/debris was resuspended in 1 mL water. Calibration standard curves were prepared using final concentrations of 0.03 to 400 μmol/L by spiking 20 μL of serial working standard solutions into 180 μL of lysed, untreated ACE2-A549 cells. For protein precipitation, trichloroacetic acid was added followed by centrifugation at 13,000 rpm for 10 min. The pH-neutralized extract was injected into our UPLC-MS/MS systems (see above).

The UPLC separation of EIDD-1931, NHC-TP, cytidine, and CYT-TP was performed using the same UPLC conditions as described for EIDD-1931 above. The multiple reaction monitoring conditions were 257.9 to 125.9 *m/z* (for quantification) and 257.9 to 168.0 *m/z* (as qualifier) for EIDD-1931, 498.0 to 159.0 *m/z* (for quantification) and 498.0 to 400.0 *m/z* (as qualifier) for NHC-TP, 241.9 to 110.0 *m/z* (for quantification) and 241.9 to 152.1 *m/z* (as qualifier) for cytidine, 247.0 to 110.9 *m/z* (for quantification) and 247.0 to 153.8 *m/z* (as qualifier) for isotope-labeled (^13^C_5_) cytidine, 481.9 to 158.9 *m/z* (for quantification) and 481.9 to 401.8 *m/z* (as qualifier) for CYT-TP, 486.9 to 158.9 *m/z* (for quantification), and 486.9 to 406.8 *m/z* (as qualifier) for isotope-labeled (^13^C_5_) CYT-TP.

Precision was 5.9%, 16%, 0.3%, 6.7%, and 8.1%, and accuracy was −0.5%, 2.3%, 7.7%, 13.5%, and −8.2% at 0.06 (LLOQ), 0.3, 3, 30, and 100 μmol/L for EIDD-1931. Precision was 2.2%, 3.2%, 0.7%, 0.6%, and 10.3%, and accuracy was −3.0%, 3.3%, −1.0%, 5.0%, and −0.3% at 0.1 μmol/L (LLOQ), 0.6, 3, 30, and 100 μmol/L of NHC-TP. Precision was 16.2%, 0.5%, 3.2%, 4.1%, and 7.7%, and accuracy was −1.0,% 1.5%, −7.0%, 5.3%, and −5.3% at 0.03 μmol/L (LLOQ), 0.1, 3, 30 and 100 μmol/L of isotope-labeled (^13^C_5_) cytidine. Precision was 4.9%, 7.9%, 0.2%, 1.0%, and 9.3%, and accuracy was 3.0%, −3.3%, 3.8%, 3.0%, and 0.6% at 0.06 μmol/L (LLOQ), 0.3, 3, 30 and 100 μmol/L of isotope-labeled (^13^C_5_) CYT-TP.

### Data availability.

The following reagent was deposited by the Centers for Disease Control and Prevention and obtained through BEI Resources, NIAID, NIH, SARS-Related Coronavirus 2, Isolate USA-WA1/2020, NR-52281.

## References

[1] Baden LR, El Sahly HM, Essink B, Kotloff K, Frey S, Novak R, Diemert D, Spector SA, Rouphael N, Creech CB, McGettigan J, Khetan S, Segall N, Solis J, Brosz A, Fierro C, Schwartz H, Neuzil K, Corey L, Gilbert P, Janes H, Follmann D, Marovich M, Mascola J, Polakowski L, Ledgerwood J, Graham BS, Bennett H, Pajon R, Knightly C, Leav B, Deng W, Zhou H, Han S, Ivarsson M, Miller J, Zaks T, COVE Study Group. 2021. Efficacy and safety of the mRNA-1273 SARS-CoV-2 vaccine. N Engl J Med 384:403–416. doi:10.1056/NEJMoa2035389. 33378609 PMC7787219

[2] Polack FP, Thomas SJ, Kitchin N, Absalon J, Gurtman A, Lockhart S, Perez JL, Pérez Marc G, Moreira ED, Zerbini C, Bailey R, Swanson KA, Roychoudhury S, Koury K, Li P, Kalina WV, Cooper D, Frenck RW, Hammitt LL, Türeci Ö, Nell H, Schaefer A, Ünal S, Tresnan DB, Mather S, Dormitzer PR, Şahin U, Jansen KU, Gruber WC, C4591001 Clinical Trial Group. 2020. Safety and efficacy of the BNT162b2 mRNA COVID-19 vaccine. N Engl J Med 383:2603–2615. doi:10.1056/NEJMoa2034577. 33301246 PMC7745181

[3] Khan YH, Mallhi TH, Alotaibi NH, Alzarea AI, Alanazi AS, Tanveer N, Hashmi FK. 2020. Threat of COVID-19 vaccine hesitancy in pakistan: the need for measures to neutralize misleading narratives. Am J Trop Med Hyg 103:603–604. doi:10.4269/ajtmh.20-0654. 32588810 PMC7410483

[4] Xiao X, Wong RM. 2020. Vaccine hesitancy and perceived behavioral control: a meta-analysis. Vaccine 38:5131–5138. doi:10.1016/j.vaccine.2020.04.076. 32409135

[5] Magadmi RM, Kamel FO. 2021. Beliefs and barriers associated with COVID-19 vaccination among the general population in Saudi Arabia. BMC Public Health 21:1438. doi:10.1186/s12889-021-11501-5. 34289817 PMC8294288

[6] Bergwerk M, Gonen T, Lustig Y, Amit S, Lipsitch M, Cohen C, Mandelboim M, Levin EG, Rubin C, Indenbaum V, Tal I, Zavitan M, Zuckerman N, Bar-Chaim A, Kreiss Y, Regev-Yochay G. 2021. COVID-19 breakthrough infections in vaccinated health care workers. N Engl J Med 385:1474–1484. doi:10.1056/NEJMoa2109072. 34320281 PMC8362591

[7] Li Q, Nie J, Wu J, Zhang L, Ding R, Wang H, Zhang Y, Li T, Liu S, Zhang M, Zhao C, Liu H, Nie L, Qin H, Wang M, Lu Q, Li X, Liu J, Liang H, Shi Y, Shen Y, Xie L, Zhang L, Qu X, Xu W, Huang W, Wang Y. 2021. SARS-CoV-2 501Y.V2 variants lack higher infectivity but do have immune escape. Cell 184:2362–2371.e9. doi:10.1016/j.cell.2021.02.042. 33735608 PMC7901273

[8] McCallum M, Walls AC, Sprouse KR, Bowen JE, Rosen LE, Dang HV, De MA, Franko N, Tilles SW, Logue J, Miranda MC, Ahlrichs M, Carter L, Snell G, Pizzuto MS, Chu HY, Van Voorhis WC, Corti D, Veesler D. 2021. Molecular basis of immune evasion by the Delta and Kappa SARS-CoV-2 variants. Science 374:1621–1626. doi:10.1126/science.abl8506. 34751595 PMC12240541

[9] Autran B, Carcelain G, Li TS, Blanc C, Mathez D, Tubiana R, Katlama C, Debre P, Leibowitch J. 1997. Positive effects of combined antiretroviral therapy on CD4+ T cell homeostasis and function in advanced HIV disease. Science 277:112–116. doi:10.1126/science.277.5322.112. 9204894

[10] Mizokami M, Yokosuka O, Takehara T, Sakamoto N, Korenaga M, Mochizuki H, Nakane K, Enomoto H, Ikeda F, Yanase M, Toyoda H, Genda T, Umemura T, Yatsuhashi H, Ide T, Toda N, Nirei K, Ueno Y, Nishigaki Y, Betular J, Gao B, Ishizaki A, Omote M, Mo H, Garrison K, Pang PS, Knox SJ, Symonds WT, McHutchison JG, Izumi N, Omata M. 2015. Ledipasvir and sofosbuvir fixed-dose combination with and without ribavirin for 12 weeks in treatment-naive and previously treated Japanese patients with genotype 1 hepatitis C: an open-label, randomised, phase 3 trial. Lancet Infect Dis 15:645–653. doi:10.1016/S1473-3099(15)70099-X. 25863559

[11] Carrat F, Duval X, Tubach F, Mosnier A, van der Werf S, Tibi A, Blanchon T, Leport C, Flahault A, Mentre F, BIVIR study group. 2012. Effect of oseltamivir, zanamivir or oseltamivir-zanamivir combination treatments on transmission of influenza in households. Antivir Ther 17:1085–1090. doi:10.3851/IMP2128. 22910171

[12] Hayden FG, Sugaya N, Hirotsu N, Lee N, de Jong MD, Hurt AC, Ishida T, Sekino H, Yamada K, Portsmouth S, Kawaguchi K, Shishido T, Arai M, Tsuchiya K, Uehara T, Watanabe A, Baloxavir Marboxil Investigators Group. 2018. Baloxavir marboxil for uncomplicated influenza in adults and adolescents. N Engl J Med 379:913–923. doi:10.1056/NEJMoa1716197. 30184455

[13] Gentile I, Maraolo AE, Buonomo AR, Zappulo E, Borgia G. 2015. The discovery of sofosbuvir: a revolution for therapy of chronic hepatitis C. Expert Opin Drug Discov 10:1363–1377. doi:10.1517/17460441.2015.1094051. 26563720

[14] Tong X, Kwong AD. 2014. Barrier to resistance: lessons from 2 direct-acting hepatitis C virus inhibitors, MK-5172 and Sofosbuvir. Clin Infect Dis 59:1675–1677. doi:10.1093/cid/ciu700. 25266288

[15] Takashita E, Ejima M, Ogawa R, Fujisaki S, Neumann G, Furuta Y, Kawaoka Y, Tashiro M, Odagiri T. 2016. Antiviral susceptibility of influenza viruses isolated from patients pre- and post-administration of favipiravir. Antiviral Res 132:170–177. doi:10.1016/j.antiviral.2016.06.007. 27321665

[16] Julander JG, Siddharthan V, Evans J, Taylor R, Tolbert K, Apuli C, Stewart J, Collins P, Gebre M, Neilson S, Van WA, Lee YM, Sheridan WP, Morrey JD, Babu YS. 2017. Efficacy of the broad-spectrum antiviral compound BCX4430 against Zika virus in cell culture and in a mouse model. Antiviral Res 137:14–22. doi:10.1016/j.antiviral.2016.11.003. 27838352 PMC5215849

[17] McLeod GX, Hammer SM. 1992. Zidovudine: five years later. Ann Intern Med 117:487–501. doi:10.7326/0003-4819-117-6-487. 1503352

[18] Eyer L, Nencka R, de Clercq E, Seley-Radtke K, Růžek D. 2018. Nucleoside analogs as a rich source of antiviral agents active against arthropod-borne flaviviruses. Antivir Chem Chemother 26:2040206618761299–2040206618761223. doi:10.1177/2040206618761299. 29534608 PMC5890575

[19] Pan H, Peto R, Henao-Restrepo AM, Preziosi MP, Sathiyamoorthy V, Abdool KQ, Alejandria MM, Hernandez GC, Kieny MP, Malekzadeh R, Murthy S, Reddy KS, Roses PM, Abi HP, Ader F, Al-Bader AM, Alhasawi A, Allum E, Alotaibi A, Alvarez-Moreno CA, Appadoo S, Asiri A, Aukrust P, Barratt-Due A, Bellani S, Branca M, Cappel-Porter HBC, Cerrato N, Chow TS, Como N, Eustace J, Garcia PJ, Godbole S, Gotuzzo E, Griskevicius L, Hamra R, Hassan M, Hassany M, Hutton D, Irmansyah I, Jancoriene L, Kirwan J, Kumar S, Lennon P, Lopardo G, Lydon P, Magrini N, Maguire T, Manevska S, Manuel O, WHO Solidarity Trial Consortium. 2021. Repurposed antiviral drugs for COVID-19 - Interim WHO solidarity trial results. N Engl J Med 384:497–511. 33264556 10.1056/NEJMoa2023184 PMC7727327

[20] Hassanipour S, Arab-Zozani M, Amani B, Heidarzad F, Fathalipour M, Martinez-de-Hoyo R. 2021. The efficacy and safety of Favipiravir in treatment of COVID-19: a systematic review and meta-analysis of clinical trials. Sci Rep 11:11022. doi:10.1038/s41598-021-90551-6. 34040117 PMC8155021

[21] Apaydin CB, Cinar G, Cihan-Ustundag G. 2021. Small-molecule antiviral agents in ongoing clinical trials for COVID-19. Curr Drug Targets 22:1986–2005. doi:10.2174/1389450122666210215112150. 33588727

[22] Gottlieb RL, Vaca CE, Paredes R, Mera J, Webb BJ, Perez G, Oguchi G, Ryan P, Nielsen BU, Brown M, Hidalgo A, Sachdeva Y, Mittal S, Osiyemi O, Skarbinski J, Juneja K, Hyland RH, Osinusi A, Chen S, Camus G, Abdelghany M, Davies S, Behenna-Renton N, Duff F, Marty FM, Katz MJ, Ginde AA, Brown SM, Schiffer JT, Hill JA. 2022. Early remdesivir to prevent progression to severe COVID-19 in outpatients. N Engl J Med 386:305–315. doi:10.1056/NEJMoa2116846. 34937145 PMC8757570

[23] Gilead Sciences. 2022. FDA Approves Veklury (remdesivir) for the treatment of non-hospitalized patients at high risk for COVID-19 disease progression. https://www.gilead.com/news-and-press/press-room/press-releases/2022/1/fda-approves-veklury-remdesivir-for-the-treatment-of-nonhospitalized-patients-at-high-risk-for-covid19-disease-progression. Accessed January 23, 2022.

[24] Sheahan TP, Sims AC, Zhou S, Graham RL, Pruijssers AJ, Agostini ML, Leist SR, Schafer A, Dinnon KH, III, Stevens LJ, Chappell JD, Lu X, Hughes TM, George AS, Hill CS, Montgomery SA, Brown AJ, Bluemling GR, Natchus MG, Saindane M, Kolykhalov AA, Painter G, Harcourt J, Tamin A, Thornburg NJ, Swanstrom R, Denison MR, Baric RS. 2020. An orally bioavailable broad-spectrum antiviral inhibits SARS-CoV-2 in human airway epithelial cell cultures and multiple coronaviruses in mice. Sci Transl Med 12:eabb5883. doi:10.1126/scitranslmed.abb5883. 32253226 PMC7164393

[25] Jayk BA, Gomes da Silva MM, Musungaie DB, Kovalchuk E, Gonzalez A, Delos RV, Martin-Quiros A, Caraco Y, Williams-Diaz A, Brown ML, Du J, Pedley A, Assaid C, Strizki J, Grobler JA, Shamsuddin HH, Tipping R, Wan H, Paschke A, Butterton JR, Johnson MG, De AC, MOVe-OUT Study Group. 2022. Molnupiravir for oral treatment of COVID-19 in nonhospitalized patients. N Engl J Med 386:509–520. doi:10.1056/NEJMoa2116044. 34914868 PMC8693688

[26] Arribas JR, Bhagani S, Lobo SM, Khaertynova I, Mateu L, Fishchuk R, Park WY, Hussein K, Kim S-W, Ghosn J. 2022. Randomized trial of molnupiravir or placebo in patients hospitalized with COVID-19. N Engl J Med Evidence 1:EVIDoa2100044. doi:10.1056/EVIDoa2100044. 38319178

[27] Painter WP, Holman W, Bush JA, Almazedi F, Malik H, Eraut NCJE, Morin MJ, Szewczyk LJ, Painter GR. 2021. Human safety, tolerability, and pharmacokinetics of molnupiravir, a novel broad-spectrum oral antiviral agent with activity against SARS-CoV-2. Antimicrob Agents Chemother 65:e02428-20. doi:10.1128/AAC.02428-20. 33649113 PMC8092915

[28] Humeniuk R, Mathias A, Cao H, Osinusi A, Shen G, Chng E, Ling J, Vu A, German P. 2020. Safety, tolerability, and pharmacokinetics of remdesivir, an antiviral for treatment of COVID-19, in healthy subjects. Clin Transl Sci 13:896–906. doi:10.1111/cts.12840. 32589775 PMC7361781

[29] Pfizer. 2021. Fact sheet for healthcare providers: emergency use authorization for paxlovid. https://www.fda.gov/media/155050/download. Accessed January 29, 2021.

[30] Merck. 2021. Merck and Ridgeback's molnupiravir receives U.S. FDA emergency use authorization for the treatment of high-risk adults with mild to moderate COVID-19. https://www.merck.com/news/merck-and-ridgebacks-molnupiravir-receives-u-s-fda-emergency-use-authorization-for-the-treatment-of-high-risk-adults-with-mild-to-moderate-covid-19/. Accessed January 23, 2022.

[31] Treanor JJ, Hayden FG, Vrooman PS, Barbarash R, Bettis R, Riff D, Singh S, Kinnersley N, Ward P, Mills RG. 2000. Efficacy and safety of the oral neuraminidase inhibitor oseltamivir in treating acute influenza: a randomized controlled trial. US Oral Neuraminidase Study Group. JAMA 283:1016–1024. doi:10.1001/jama.283.8.1016. 10697061

[32] Hayden FG, Osterhaus AD, Treanor JJ, Fleming DM, Aoki FY, Nicholson KG, Bohnen AM, Hirst HM, Keene O, Wightman K. 1997. Efficacy and safety of the neuraminidase inhibitor zanamivir in the treatment of influenzavirus infections. GG167 Influenza Study Group. N Engl J Med 337:874–880. doi:10.1056/NEJM199709253371302. 9302301

[33] Fry AM, Goswami D, Nahar K, Sharmin AT, Rahman M, Gubareva L, Azim T, Bresee J, Luby SP, Brooks WA. 2014. Efficacy of oseltamivir treatment started within 5 days of symptom onset to reduce influenza illness duration and virus shedding in an urban setting in Bangladesh: a randomised placebo-controlled trial. Lancet Infect Dis 14:109–118. doi:10.1016/S1473-3099(13)70267-6. 24268590

[34] Haas DW, Arathoon E, Thompson MA, de Jesus PR, Gallant JE, Uip DE, Currier J, Noriega LM, Lewi DS, Uribe P, Benetucci L, Cahn P, Paar D, White AC, Jr., Collier AC, Ramirez-Ronda CH, Harvey C, Chung MO, Mehrotra D, Chodakewitz J, Nguyen BY, Protocol 054/069 Study Teams. 2000. Comparative studies of two-times-daily versus three-times-daily indinavir in combination with zidovudine and lamivudine. AIDS 14:1973–1978. doi:10.1097/00002030-200009080-00013. 10997402

[35] Lawitz E, Mangia A, Wyles D, Rodriguez-Torres M, Hassanein T, Gordon SC, Schultz M, Davis MN, Kayali Z, Reddy KR, Jacobson IM, Kowdley KV, Nyberg L, Subramanian GM, Hyland RH, Arterburn S, Jiang D, McNally J, Brainard D, Symonds WT, McHutchison JG, Sheikh AM, Younossi Z, Gane EJ. 2013. Sofosbuvir for previously untreated chronic hepatitis C infection. N Engl J Med 368:1878–1887. doi:10.1056/NEJMoa1214853. 23607594

[36] Lawitz E, Sulkowski MS, Ghalib R, Rodriguez-Torres M, Younossi ZM, Corregidor A, DeJesus E, Pearlman B, Rabinovitz M, Gitlin N, Lim JK, Pockros PJ, Scott JD, Fevery B, Lambrecht T, Ouwerkerk-Mahadevan S, Callewaert K, Symonds WT, Picchio G, Lindsay KL, Beumont M, Jacobson IM. 2014. Simeprevir plus sofosbuvir, with or without ribavirin, to treat chronic infection with hepatitis C virus genotype 1 in non-responders to pegylated interferon and ribavirin and treatment-naive patients: the COSMOS randomised study. Lancet 384:1756–1765. doi:10.1016/S0140-6736(14)61036-9. 25078309

[37] Ray AS, Fordyce MW, Hitchcock MJ. 2016. Tenofovir alafenamide: a novel prodrug of tenofovir for the treatment of human immunodeficiency virus. Antiviral Res 125:63–70. doi:10.1016/j.antiviral.2015.11.009. 26640223

[38] Makino S, Fujiwara K, Lai MM. 1987. Defective interfering particles of coronavirus. Adv Exp Med Biol 218:187–195. doi:10.1007/978-1-4684-1280-2_23. 3434436

[39] Crumpton WM, Dimmock NJ, Minor PD, Avery RJ. 1978. The RNAs of defective-interfering influenza virus. Virology 90:370–373. doi:10.1016/0042-6822(78)90322-7. 726256

[40] Liu Y, Yan LM, Wan L, Xiang TX, Le A, Liu JM, Peiris M, Poon LLM, Zhang W. 2020. Viral dynamics in mild and severe cases of COVID-19. Lancet Infect Dis 20:656–657. doi:10.1016/S1473-3099(20)30232-2. 32199493 PMC7158902

[41] Singanayagam A, Hakki S, Dunning J, Madon KJ, Crone MA, Koycheva A, Derqui-Fernandez N, Barnett JL, Whitfield MG, Varro R, Charlett A, Kundu R, Fenn J, Cutajar J, Quinn V, Conibear E, Barclay W, Freemont PS, Taylor GP, Ahmad S, Zambon M, Ferguson NM, Lalvani A. 2021. Community transmission and viral load kinetics of the SARS-CoV-2 delta (B.1.617.2) variant in vaccinated and unvaccinated individuals in the UK: a prospective, longitudinal, cohort study. Lancet Infect Dis 21:183–195. doi:10.1016/S1473-3099(21)00648-4. PMC8554486 34756186

[42] Alishaq M, Nafady-Hego H, Jeremijenko A, Al Ajmi JA, Elgendy M, Vinoy S, Fareh SB, Veronica PJ, Nooh M, Alanzi N, Kaleeckal AH, Latif AN, Coyle P, Elgendy H, Abou-Samra AB, Butt AA. 2021. Risk factors for breakthrough SARS-CoV-2 infection in vaccinated healthcare workers. PLoS One 16:e0258820. doi:10.1371/journal.pone.0258820. 34653228 PMC8519462

[43] Mossel EC, Huang C, Narayanan K, Makino S, Tesh RB, Peters CJ. 2005. Exogenous ACE2 expression allows refractory cell lines to support severe acute respiratory syndrome coronavirus replication. J Virol 79:3846–3850. doi:10.1128/JVI.79.6.3846-3850.2005. 15731278 PMC1075706

[44] Franco EJ, Warfield KL, Brown AN. 2022. UV-4B potently inhibits replication of multiple SARS-CoV-2 strains in clinically relevant human cell lines. Front Biosci (Landmark Ed) 27:3. doi:10.31083/j.fbl2701003. 35090308

[45] Brown AN, Strobel G, Hanrahan KC, Sears J. 2021. Antiviral activity of the propylamylatin(TM) formula against the novel coronavirus SARS-CoV-2 in vitro using direct injection and gas assays in virus suspensions. Viruses 13:415. doi:10.3390/v13030415. 33807769 PMC7999574

[46] McSharry JJ, Weng Q, Brown A, Kulawy R, Drusano GL. 2009. Prediction of the pharmacodynamically linked variable of oseltamivir carboxylate for influenza A virus using an in vitro hollow-fiber infection model system. Antimicrob Agents Chemother 53:2375–2381. doi:10.1128/AAC.00167-09. 19364864 PMC2687202

[47] Brown AN, Adams JR, Baluya DL, Drusano GL. 2015. Pharmacokinetic determinants of virological response to raltegravir in the in vitro pharmacodynamic hollow-fiber infection model system. Antimicrob Agents Chemother 59:3771–3777. doi:10.1128/AAC.00469-15. 25870053 PMC4468687

