# Supplementary material for: Why Molnupiravir Fails in Hospitalized Patients
Source: mBio. 2022 Nov 14;13(6):e02916-22. doi: 10.1128/mbio.02916-22 (PMC9765607; doi:10.1128/mbio.02916-22)
Supplement: TABLE S1 [file mbio.02916-22-s0003.docx]

**Table S1. Intracellular concentrations of EIDD-1931 in SARS-CoV-2-infected ACE2-expressing A549 cells.**

| **Time post-inoculation**  **(h)** | **Concentration EIDD-1931 (molecules/cell)** | | | | | | | |
| --- | --- | --- | --- | --- | --- | --- | --- | --- |
|  | **0.648 µg/ml** | | | | **3.24 µg/ml** | | | |
|  | **Tx@0h** | **Tx@24h** | **Tx@48h** | **Tx@72h** | **Tx@0h** | **Tx@24h** | **Tx@48h** | **Tx@72h** |
| **24** | 1.98E+05 | N.D.^a^ | N.D | N.D | 1.04E+06 | N.D | N.D | N.D |
| **48** | 5.04E+05 | 1.92E+05 | N.D | N.D | 5.13E+05 | 4.58E+05 | N.D | N.D |
| **72** | 2.92E+04 | 7.58E+04 | 6.78E+04 | N.D | 2.38E+05 | 2.80E+05 | 1.03E+06 | N.D |
| **96** | 1.09E+04 | 8.22E+04 | < L.O.D.^b^ | 1.88E+05 | 1.35E+05 | 2.38E+05 | 3.02E+04 | 3.75E+05 |
| **120** | < L.O.D. | 2.55E+05 | < L.O.D. | < L.O.D. | 2.70E+05 | 2.90E+05 | 5.12E+04 | 2.36E+05 |

^a^ N.D. = not done

^b^ < L.O.D. = less than limit of detection. The limit of detection for this ultra-performance liquid chromatography-tandem mass spectrometry assay is 0.016 µg/mL.
