# Supplementary figures and images for: Why Molnupiravir Fails in Hospitalized Patients
Source: mBio. 2022 Nov 14;13(6):e02916-22. doi: 10.1128/mbio.02916-22 (PMC9765607; doi:10.1128/mbio.02916-22)

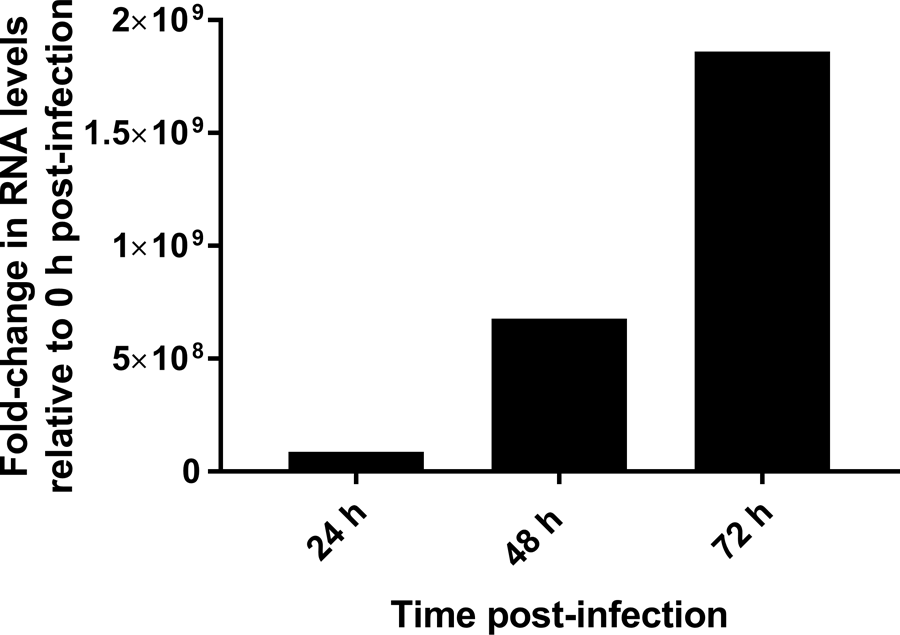

Supplement: FIG S1 [file mbio.02916-22-s0001.tif]
