# Supplementary material for: Why Molnupiravir Fails in Hospitalized Patients
Source: mBio. 2022 Nov 14;13(6):e02916-22. doi: 10.1128/mbio.02916-22 (PMC9765607; doi:10.1128/mbio.02916-22)
Supplement: TEXT S1 [file mbio.02916-22-s0002.docx]

**Supplementary methods.** Cellular RNA was isolated from 1x10^7^ infected ACE2-A549 cells using Qiagen’s RNeasy mini kit (Qiagen). Extracts were frozen at -80°C until quantitative real-time reverse transcription-PCR (qRT-PCR) was performed on samples. Viral RNA was amplilfied using TaqMan Fast Virus 1 Step Master Mix (Applied Biosystems) and a 2019-nCoV CDC EUA Authorized qPCR primer/probe set targeting the N1 region of the SARS-CoV-2 nucleocapsid gene (Integrated DNA Technologies): primers 5’-GACCCCAAAATCAGCGAAAT-3’ (forward, 500 nM), and 5’-TCTGGTTACTGCCAGTTGAATCTG-3’ (reverse, 500 nM), and probe 5’-FAM-ACCCCGCAT/ZEN/TACGTTTGGTGGACC-3IABkFQ (125 nM). The thermal cycling conditions were 50°C for 5 min, 95°C for 20 seconds, followed by 40 cycles of 95°C for 3 seconds and 60°C for 30 seconds. Reactions were analyzed using the ViiA7 Real Time PCR System (Applied Biosystems). Fold changes in viral RNA levels between time points were calculated using the delta C_T_ method.
